# Changes in Ventilatory Support Requirements of Spinal Muscular Atrophy (SMA) Patients Post Gene-Based Therapies

**DOI:** 10.3390/children9081207

**Published:** 2022-08-11

**Authors:** Panagiota Panagiotou, Christina Kanaka-Gantenbein, Athanasios G. Kaditis

**Affiliations:** 1 Department on Pediatric Respiratory Medicine, Evelina London Children’s Hospital, London SE1 7EH, UK; 2 Division of Pediatric Pulmonology and Sleep Disorders Laboratory, First Department of Pediatrics, Agia Sofia Children’s Hospital, Medical School, National and Kapodistrian University of Athens, 11527 Athens, Greece

**Keywords:** gene-based therapies, noninvasive ventilation, Nusinersen, Onasemnogene abeparvovec, respiratory care, spinal muscular atrophy

## Abstract

Spinal muscular atrophy (SMA) is a genetic neuromuscular disease resulting in global muscular weakness and, frequently, in respiratory failure and premature death. Gene-based therapies like Nusinersen are now available for patients with SMA. The aim of this review was to assess in “real world” studies, whether novel treatments would have a positive impact on the mechanical ventilatory support requirements of SMA patients, already initiated on ventilatory support prior to treatment administration. A literature search was performed in Pubmed using multiple combinations of MESH terms and the snowball procedure. A total of 14 publications were discussed in this review. Considering all patients included in the published studies who were on ventilatory support and were treated with Nusinersen, 13/172 (7.5%) had reduced needs for ventilatory support, 1/172 (0.6%) did not need ventilation post-treatment, and 122/172 (70.9%) were maintained on the same ventilator settings. Moreover, 2/41 (4.9%) children who were offered gene therapy had no need for further ventilatory support and 12/41 (29.2%) had reduced requirements. In conclusion, available evidence suggests that among children with SMA, who are on mechanical respiratory support either noninvasively or via tracheostomy at the time of gene-based treatment, only a few will be weaned off the ventilator or have reduced ventilator needs per 24 h. Children will usually require the same level of support as before treatment.

## 1. Introduction

Spinal muscular atrophy (SMA) is one of the well-studied neuromuscular diseases with genetic origin and a prevalence of 1/6000 to 1/11,000 [1,2]. It is an autosomal recessive disorder leading to degeneration of the anterior horn cells of the spinal cord and of the motor neurons in the lower brainstem [3]. The disease is caused by mutation in the survival motor neuron (SMN) 1 gene, which encodes the SMN protein in humans [4]. SMA is classified according to the severity of clinical manifestations and the age of onset of symptoms, which varies from neonatal to adult life [1,5]. Severity of the disease correlates with the copy numbers of a functional protein transcribed by an almost identical gene named SMN2 (disease-modifying gene), although it does not absolutely predict SMA progression [6,7,8,9]. SMA leads to global, severe muscular weakness affecting especially free-body movements and respiration [1,9]. However, it does not have an impact on higher cortical functions [10,11]. The previous classification of SMA in Types 1, 2, and 3 was recently updated in the literature with a more detailed one [1,7]. In Table 1, the five types of SMA are presented along with their clinical characteristics [7,12,13].

The above table refers to the clinical course of SMA, when “letting nature takes its course”. Research has shown that with the provision of supportive measures to SMA1 patients, such as respiratory and feeding support, the survival probability after the second birthday is higher and the survival time without respiratory insufficiency increases [14,15].

SMA is more than a motor neuron disease though. The expression of SMN protein affects many tissues and organs like muscles, kidney, spleen, bone, connective tissue, liver, and vasculature and the possible associated adverse consequences are under research [16,17,18,19,20,21].

### 1.1. Pathophysiology of Respiratory Disease in SMA

Respiratory disease is a common cause of morbidity, disability, and mortality among patients with SMA [22,23]. SMA patients develop inspiratory and expiratory muscle weakness, which primarily ends up in an ineffective cough, contributing to frequent respiratory infections [13]. Gastroesophageal reflux and bulbar weakness, which affect individuals with SMA1, will contribute to respiratory infections [13,24]. Intercostal muscles are weaker than the diaphragm, resulting in chronic thoraco-abdominal asynchrony that promotes chest wall deformities in young children, such as “bell-shaped” chest and pectus excavatum [6,13,24]. Dysfunction of the diaphragm and other respiratory muscles and the associated reduced inspiratory capacity, ineffective cough, as well as poor swallowing, coordination, and management of secretions have a detrimental effect on respiratory health [23].

Acutely, SMA patients may manifest respiratory deterioration due to their inability to cope with respiratory infections, aspiration episodes, or procedures requiring general anesthesia, which would require invasive ventilation, due to increased respiratory load, which can be compensated up to a point [24,25]. Beyond this point, hypoventilation develops leading to hypercapnic respiratory failure [26]. Thus, SMA patients require intubation and ventilation and have difficulty being weaned off respiratory support [27].

Chronic respiratory failure may also develop slowly, as the illness progresses over time. Lungs are affected by microatelectasis and mucus plugging, which in combination with respiratory muscle weakness, lead to increased respiratory rate, chronic hypoxemia, and hypoventilation [26].

Due to the physiological changes affecting the respiratory system of even healthy individuals during sleep, namely decreased functional residual capacity, hypotonia of the respiratory muscles, and increased upper airway resistance, patients with SMA compensating for the exaggerated respiratory load during the day may not be able to do so at night [1,28]. Airway clearance techniques with nebulizers and insufflation/exsufflation devices prevent mucus plugging and recurrent hospital admissions for respiratory infections [28].

Sleep-disordered breathing (SDB) in patients with SMA is characterized by hypoventilation with a variable component of intermittent upper airway obstruction (obstructive and mixed apneas and hypopneas) and hypoxia which can be the result of hypoventilation or mucus plugging [1,10,13,25]. With progression of the disease, daytime hypoventilation and hypercapnia may occur [1]. Polysomnography is the gold standard method for the early diagnosis of SDB in children with neuromuscular disorders and provides information on sleep efficacy, sleep quality, and architecture in addition to respiratory events and gas exchange abnormalities [1,29]. Oximetry with capnography (oxycapnography) is an alternative for the diagnosis of SDB and monitoring of the efficacy of noninvasive ventilation (NIV) in children with neuromuscular disorders [1,26,30]. Children with SMA and respiratory failure usually have increased energy needs and somatic growth failure [26].

### 1.2. NIV and Respiratory Supportive Care

Up until recently, the treatment for SMA patients was only supportive, having as a goal the reduction of the work of breathing. This was achieved by ventilatory support and airway clearance [24,31]. SDB and nocturnal and/or diurnal hypoventilation were treated with long-term ventilation [23]. Initially, the ventilatory support was mostly invasive—via tracheostomy—but over the last 10–20 years and with the evolution of new technologies, long-term ventilation has been provided via NIV interfaces [31,32]. NIV, apart from providing the necessary respiratory support, is shown to prevent pectus excavatum and promote lung and chest wall growth [33]. In patients with SMA1, hypoventilation is not always obvious and for this reason some centers have developed algorithms for NIV initiation. For example, Edel et al. have assessed SMA patients using an algorithm that is taking into consideration frequency of respiratory exacerbations, physical examination findings, sleep study results, and nutritional status [34]. The goals of long-term NIV are improvement of sleep quality and quality of life as well as prolonging survival

Nevertheless, even in cases where supportive measures are taken early in life (ventilator support, airway clearance techniques) according to standards of care, there is a progressive decline in respiratory function [32]. Recently, two gene-based disease-modifying treatments have been approved and introduced in the care of patients with SMA. Nusinersen (Spinraza, Biogen, Cambridge, MA, USA), was the first gene-based disease-modifying therapy approved in the USA in December 2016, and in June 2017 in Europe [7]. Nusinersen is an antisense oligonucleotide that is administered intrathecally, following a detailed protocol, and modulates the SMN2 pre-mRNA splicing and thus the synthetized protein levels in the central nervous system supporting motor neuron development [4,5]. Onasemnogene abeparvovec (Zolgensma) is a gene replacement therapy, involving a one-time intravenous infusion of an adeno-associated virus serotype 9 (AAV9), which delivers the *SMN* transgene under a ubiquitous promoter into target cells. Onasemnogene abeparvovec was approved in May 2019, in the USA and in May 2020 in Europe for children younger than 2 years with SMA and with three or fewer SMN copies [22]. Risdiplam (Evrysdi) is a small molecule, SMN2-splicing modifier, administered daily orally, approved in the USA in August 2020 and in Europe in March 2021 [35].

Hence, the aim of this review was to assess the potential decrease in the need for ventilatory support among SMA patients already initiated on respiratory support prior to treatment with one or both of the gene-based therapies.

## 2. Materials and Methods

We performed a comprehensive literature search on the PubMed database using the following combinations of MESH-terms: (“Spinal Muscular Atrophy“ AND “gene therapy” OR “nasemnogene abeparvovec-AVXS-101” AND respiratory), (“spinal muscular atrophy” AND “Nusinersen” AND respiratory), and (“spinal muscular atrophy” AND Risdiplam AND respiratory). We followed the “snowball procedure”, meaning that the references of the included articles were also searched for potentially relevant articles. The literature search covered the past 5 years (last search on 10 April 2022) and referred to the pediatric population (<18 years old).

Articles were eligible for inclusion if they reported “real world” studies and provided information on NIV support before initiation with gene-based therapy and for at least 4 months while on treatment. Case reports were also included. We have excluded studies published in languages other than English and studies involving patients with diagnosis other than SMA requiring NIV. Moreover, we excluded those articles published as part of clinical trials, because these types of studies measure the internal validity of the treatment interventions while we aimed to check the external validity and therefore the generalization of the results of the treatments across different situations and times.

The primary outcome was the percentage of patients who were able to wean off or to reduce the time on the ventilator per 24 h after receiving one of the novel therapies for SMA. Criteria for weaning mechanical respiratory support and the role of sleep studies to that procedure were also discussed.

## 3. Results

### 3.1. Study Selection

Under the algorithm (“Spinal Muscular Atrophy” AND Nusinersen AND respiratory), 83 studies were retrieved and screened. We excluded 62 studies based on the abstracts and reviewed 21 studies in full text. Of these 21 publications, 10 met our inclusion criteria. Under the algorithm (“Spinal Muscular Atrophy“ AND “gene therapy” OR “onasemnogene abeparvovec-AVXS-101” AND respiratory), 121 studies were retrieved and screened. Only 4 of them were suitable for full-text review based on the abstracts and all 4 met the inclusion criteria. Under the algorithm (“Spinal Muscular Atrophy” AND Risdiplam AND respiratory), 19 studies were retrieved. We excluded 16 based on the abstract and reviewed 3 in full text. None of which met the inclusion criteria.

### 3.2. Patients’ Characteristics

When all subjects from the 14 included studies were considered together, data were available from 255 patients with SMA who received Nusinersen and from 118 who received gene therapy. Of the 118 individuals who were treated with gene therapy, 78 had previously received Nusinersen. The author groups had wide geographical distribution: Australia, Austria, Brazil, Germany, Italy, Israel, Japan, Korea, and United States of America. The age of patients at the time they received the treatment varied appreciably. For patients receiving gene therapy, the median age of treatment was below 24 months in the included studies. The age of patients who received Nusinsersen varied widely, especially in reports involving patients with SMA3. The number of SMN copies was not mentioned in every publication. A short overview of patients’ characteristics is presented in Table 2. It should be noted that the number of patients in Table 2 refer to the total number of subjects included in the studies, meaning patients self-ventilated on air, on NIV, and on IMV at the time of receiving the treatment.

### 3.3. Nusinersen

There were several “real world” studies in the literature evaluating the motor function improvement and milestone achievements, as well as somatic growth in individuals with SMA post-Nusinersen, but only a few of them were focused on respiratory outcomes.

#### Data on NIV Support Requirements Pre- and Post-Nusinersen Therapy

Table 3 summarizes changes in ventilatory support requirements for each study. Lavie et al. and Ali et al. identified no reduction in NIV needs 24 months post-treatment [36,43]. De Holanda Mendonca et al. reported data on SMA1 patients with different duration of monitoring and it took 6–24 months to demonstrate a decrease in NIV support needs in only a minority of them [42]. Similarly, Kim et al. were able to identify diminished NIV use by only two hours post-Nusinersen in 50% of a small cohort of patients, who were on NIV at baseline [39]. In a nationwide study from Italy, Sansone et al. followed up on 88 SMA patients on NIV at baseline for a 10-month period and reduced NIV support needs were noted in only 3.4% of them, whereas in most participants no change was identified [32]. In another study from Italy, Pane et al. showed that over a period of 12 months, 2 out of 35 (5.7%) subjects with SMA required less NIV support compared to pretreatment and 11 of 35 (31.4%) had stable NIV settings [38]. Results of two additional case reports are summarized in Table 3 [40,44].

Taking under consideration all patients treated with Nusinersen in all included studies who were receiving respiratory support at baseline, a reduction in ventilator settings was noted in only 7.5% of them and unchanged settings in 70.9% during monitoring for up to 24 months post-treatment initiation [32,36,38,39,40,42,43,44].

Table 4 summarizes studies in which an association between the SMN copies and respiratory outcome post-Nusinersen treatment could be identified. A reduction of time on the ventilator was achieved in 26% of the patients with two copies of SMN whilst in 40% of patients with three copies of SMN. However, it should be noted that the total number of included patients was small.

### 3.4. Replacement of SMN1-Gene

As gene replacement therapy has been approved quite recently, there are very few studies presenting “real world” data for SMA patients who received this therapy.

#### Data on NIV Support Needs Post-Gene Therapy

Three studies and one case report have focused on the respiratory outcomes in patients who have received gene therapy and their results are summarized in Table 5. D’Silva et al. followed up 21 patients with SMA (7 of them already on ventilator support at baseline) who received gene therapy. Among those children, 19 had already received Nusinersen, including 7 patients already on NIV prior to gene-therapy administration. Stabilization in respiratory function was demonstrated in 18 patients (5 on the ventilator and 13 remained self-ventilated on air), while 2 patients required less time per 24 h on the ventilator [45]. Weiss et al. presented a multicenter study from Germany and Austria including 76 children with SMA who received gene therapy, of which 58 had been treated previously with Nusinersen [47]. Of the 76 patients, 24 were on NIV at baseline. Of those, 9 individuals required fewer hours per 24 h on the ventilator and 1 was weaned off respiratory support. No further information was given regarding how many of them were Nusinsersen naïve nor how many had increased or unchanged ventilator needs on follow-up [47]. Waldorp et al. followed up 21 children with SMA treated with gene therapy, of whom 11 had previously received Nusinersen [48]. Data on respiratory status was available for 20 of them. A total of 9 patients were on NIV prior to treatment. Of those, 1 needed decreased time of support per 24 h post-gene therapy, and 1 was weaned off NIV. Both patients had received Nusinersen previously. Seven subjects (2 of which were Nusinersen naïve) did not have any changes in the NIV settings. Of note, among the 13 patients who were not on NIV and received gene therapy, only 1 required NIV support later on [48]. Mateszanz et al. described the case of an SMA0 patient who received both Nusinersen and gene therapy while already ventilated via tracheostomy and his respiratory status remained stable on IMV at one year of life. Some improvement in bulbar function and strength of cough was reported by the family [46]. Results related to gene therapy are summarized in Table 5.

### 3.5. Criteria Used to Reduce Time on or Wean off Ventilator Support

In none of the included studies were the exact criteria of initiating ventilatory support, changing settings, or decreasing the time on the ventilator mentioned (NIV or IMV via tracheostomy). Sansone et al. used nocturnal oximetries or sleep studies to assess the need for mechanical respiratory support along with the number of respiratory infections [32]. Lavie et al. stated that abnormalities in polysomnography and chest wall hypoplasia or deformity were part of their criteria for initiating NIV, and the same modalities were applied to monitor its efficacy [43]. Polysomnography was also used to evaluate respiratory function in the study by Weiss et al. [47].

## 4. Discussion

### 4.1. NIV Support for “Treated” SMA Patients

The term “treated SMA” is a newly emerging term in the literature to describe SMA patients who have received disease-modifying therapies [41]. Review of the available studies on respiratory outcomes in children with SMA, who were on mechanical respiratory support either noninvasively or via tracheostomy at the time of treatment, revealed data for treatment with Nusinersen or Onasemnogene abeparvovec but no “real world” data could be found on Risdiplam, which seems to be an open field for future research. It is indicated that only a few of the SMA patients, already on respiratory support, will be weaned off the ventilator or will have reduction in ventilator needs per 24 h. Children will usually require the same level of support as before treatment.

Comparison of the results of our review with the results of papers on clinical trials is difficult to perform, as the observed outcomes are different. The aim of clinical trials, such as ENDEAR for Nusinersen [49] or the START study [50] for Onasemnogene abeparvovec, was to assess the event-free survival, defined as the time till death or till the use of permanent assisted ventilation (tracheostomy or ventilator support for ≥16 h per day) continuously for at least 14 or 21 days in the absence of an acute reversible event [49,50]. A study by Poland et al. and another one by Pechmann et al. have, respectively, shown significant and non-significant increase in respiratory support post-treatment with Nusinersen [51,52]. Nevertheless, in the latter study, 7% of the patients showed reduced NIV needs, and one subject was weaned off the ventilator. So, in those studies, it is not possible to accurately assess the changes in the hours of ventilator use if the duration is no longer than the cut-off limit, which differentiates permanent from partial ventilation.

Children already on IMV via tracheostomy usually have poor response to treatment, although there may be exceptions [32,37,42]. From the included studies, it is also evident that patients with severe SMA type as well as those with low neuromuscular scores, like the Hammersmith Infant Neurological Examination Score (HINE-Score), are less likely to improve from a respiratory point of view [32,42]. Aragon Gawinska et al. reported that the number of SMN copies did not influence the need for ventilator support, whereas Chen et al. suggested that children with two SMN copies required more frequent admissions and had a higher incidence of NIV initiation compared to children with three SMN copies [51,53]. In support of that, in our review, children with two SMN copies had worse outcome than those with three SMN copies. Nevertheless, further studies are necessary to assess a potential correlation of the number of SMN copies with respiratory outcomes after treatment.

Studies demonstrate that the earlier children receive treatment post-diagnosis, the higher the likelihood for reduced or no need for mechanical respiratory support [19,20,49,54]. In the study by Kim et al., patients with de-escalation of respiratory support needs were given Nusinersen within one month post-diagnosis, while in the report by De Holanda Mendonca et al., subjects with the worst respiratory course were those who received Nusinersen more than 2 years after SMA diagnosis [42]. This finding is consistent with the fact that during the first 6 months of life, degeneration of the spinal motor units in children with SMA follows a rapid course that is potentially delayed by the available novel treatments [55]. The introduction of neonatal screening for SMA in different countries would facilitate earlier diagnosis and allow earlier treatment, which could potentially lead to improved respiratory outcomes [56].

Due to the increased complexity in the care of children with SMA and the availability of novel treatments, standardization of their respiratory follow-up is imperative [57]. Polysomnography, polygraphy, or nocturnal oxy-capnometry should be part of this monitoring algorithm aiming to formulate a ventilator weaning protocol, as the course of this newly emerged “treated SMA” is not known yet [10]. Sleep studies could provide clinicians with objective respiratory parameters and facilitate treatment decisions [58]. Gene-based treatments may fill parents with hope, since they are frequently skeptical of NIV. However, the lack of long-term data on the respiratory outcomes of gene-based disease-modifying therapies needs to be communicated to them [32]. Further research is necessary to assess the effect of those treatments, either separately or as a combination, to the respiratory function and need for ventilator support of SMA patients [59]. Based on the available data, it should be made clear to the families that children with SMA will most likely need NIV and supportive treatment despite gene-modifying therapies [52,60].

In this discussion with families, the attitude of the physician towards respiratory support of SMA patients is more important than ever [15,33]. The respiratory team need to be involved as early as possible in the care of SMA patients [61]. As the decision about ventilation initiation is often a joint decision with the family, caregivers have to be aware that close respiratory monitoring is needed [15,54]. SMA registries could facilitate standardized and consistent follow-up visits and data exchange among clinicians and researchers [28]. The goal of every discussion and treatment plan should be to provide SMA patients with the best possible quality of life, which is often grossly underestimated [33].

### 4.2. SDB and Respiratory Muscle Strength in “Treated” SMA

Since Nusinersen is associated with improved muscle strength, we would expect an increase in the tone of upper airway dilator skeletal muscles, stronger cough and airway clearance as well as better support of the thoracic cage, less severe scoliosis, and better ventilation and oxygenation [40]. Banda et al. studied the inspiratory and expiratory muscle performance in children with SMA2 and demonstrated an improvement post-Nusinersen treatment [60]. Additionally, Nusinersen has been proven to have an ameliorative effect on the respiratory function of infants and children with SMA1 and on the degree of thoraco-abdominal asynchrony [62]. Nevertheless, the disease subtypes seem to play a crucial role in the response to treatment [62]. The quantification of the degree of respiratory muscle weakness over time may be helpful for monitoring the progression of respiratory muscle weakness, and it is of critical importance for evaluating the efficacy of novel therapies. Assisted cough and airway clearance techniques should continue to be offered as a measure for better respiratory health and avoidance of infections [24,56].

In a case report, treatment with Nusinersen was related to a decrease in the apnea-hypopnea index and normalization of the sleep architecture [40]. Nonetheless, more data and longer follow-up are necessary to establish the reproducibility of this finding. Patients who received gene therapy seem to have better respiratory outcomes than those treated with Nusinersen only. However, in many cases, gene therapy had been preceded by treatment with Nusinersen. Close monitoring and regular assessment of SMA patients, who have received novel treatments and they are on ventilatory support, with polysomnography is necessary to assess the effects of treatment [54].

### 4.3. Limitations

This review covers “real word” studies published in the last 5 years. We felt that this time frame would be appropriate as Nusinersen was approved in December 2016 and gene therapy and Risdiplam later. The limitations of this review reflect the limitations of the included studies. A heterogeneous group of patients is presented, with a broad age range, who started treatment at different stages of their disease and who have different numbers of SMN copies (Table 4). The included patients had respiratory failure of different severity and follow-up for variable intervals. Moreover, the indications for starting NIV at baseline were not clearly defined (e.g., as a proactive measure, due to chest deformities, nocturnal hypoventilation or recurrent respiratory infections) [63]. The respiratory follow-up plan and outcome measures were not universally standardized [37]. For example, the duration of partial ventilation per 24 h was arbitrarily defined in each study. In the study by Pane et al., partial ventilation was defined as fewer than 10 h on the ventilator, but in the study by de Holanda Mendonca et al. and Lavie et al., 16 h was used as the cut-off value [38,42,43]. Last but not least, the study selection and data abstraction were performed by a single reviewer.

## 5. Conclusions

The clinical course of SMA is well-studied, and it is known to lead to global muscular weakness, respiratory failure, and shortened life span. Results of the first “real world” studies indicate that very few children, who have already developed respiratory failure when they are offered gene-based treatment, will be weaned partially or completely off the ventilator. Further research is necessary to assess the effect of those novel treatments on the respiratory status of patients with SMA and to develop standardized longitudinal follow-up protocols. Investigations for the assessment of the ventilatory status of those patients, such as polysomnography, and evaluation of exact ventilator parameters (inspiratory/expiratory pressures, hours on the ventilator, degree of thoraco-abdominal asynchrony, shape of thorax) should be included to enable a better understanding of the pathophysiology of “treated SMA” disease. This will allow a more effective approach to the respiratory care of SMA patients in the era of gene-based disease-modifying therapies.

## Figures and Tables

**Table 1:** Spinal muscular atrophy (SMA) types.

	SMA0	SMA1	SMA2	SMA3	SMA4
Milestone achieved	None	Nonsitters	Sitters	Walkers	Normal
Time of Onset	Prenatal	<6 months	7–18 months	>18 months	Adult Onset
No. of SMN Copies	0–1	1–2	2–3	3–4	>4
Survival (“letting nature take its course”)	Most severe, fatal by age 6 months	More frequent fatal by age 2 years	Survival to adulthood	Survival to adulthood	Survival to adulthood

**Table 2:** Patients’ characteristics (with follow-up data).

Author/Year/ Country	No. of Patients	Treatment Received	Age at Treatment/Previous Treatment	Monitoring Interval
Ali et al./2019/UK [36]	11	Nusinersen	8.1 months (median)	24 months
Ogawa et al./2019/Japan [37]	1	Nusinersen	99 days	6 months
Pane et al./2019/Italy [38]	85	Nusinersen	2 months–15 years (range)	12 months
Kim et al./2020/Korea [39]	6	Nusinersen	0.5 months–98 months (range)	6 months
Kouri et al./2020/U.S.A [40]	1	Nusinersen	10 years	12 months
Sansone et al./2020/Italy [32]	109	Nusinersen	42.8 months (median)	10 months
Tiberi et al./2020/Italy [41]	1	Nusinersen	13 days	5 months
Holando Menonca et al./2021/Brazil [42]	21	Nusinersen	5 months–120 months (range)	6 months– 24 months
Lavie et al./2021/Israel [43]	19	Nusinersen	13.5 months (median)	24 months
Tanaka et al./2021/Japan [44]	1	Nusinersen	9 years 5 months/valproate	24 months
D’ Silva et al./2022/Australia [45]	21	Gene Therapy	11 months (median)/ 19 patients: Nursinersen	15 months
Mateszenz et al./2022/USA [46]	1	Gene Therapy	114 days/ Nursinersen	10 months
Weiss et al./2022/Austria and Germany [47]	76	Gene Therapy	16.8 months (median)/ 58 patients: Nursinersen	23 weeks– 83 weeks
Waldrop et al./2020/USA [48]	20	Gene Therapy	1 month–23 months (range)	4 months

**Table 3:** Changes in ventilatory support requirements following treatment with Nusinersen.

Author/Year	Reduction in NIV/IMV Support	Discontinued NIV	Unchanged NIV/IMV Support	Criteria for Ventilator Support Changes
Ali et al./2019 [36]	0	0	5/6	n/m
Ogawa et al./2019 [37]	0	0	1/1	Parameters on the ventilator
Pane et al./2019 [38]	2/35	0	11/35	n/m
Kim et al./2020 [39]	2/4	0	2/4	n/m
Kouri et al./2020 [40]	0	1/1	0	Polysomnography
Sansone et al./2020 [32]	3/88	0	75/88	Oximetry and gas exchange evaluation
Tiberi et al./2020 [41]	0	0	0/1	n/m
De HolandaMendonca et al./2021 [42]	6/21	0	14/21	n/m
Lavie et al./2021 [43]	0	0	13/15	Polysomnography Chest wall deformities
Tanaka et al./2021 [44]	0	0	1/1	n/m
**Total** **Nusinersen**	**13/172 (7.5%)**	**1/172 (0.6%)**	**122/172 (70.9%)**	**n/m**

Abbreviations: IMV: invasive mechanical ventilation via tracheostomy; NIV: noninvasive ventilation; N/M: not mentioned; SMN: survival motor neuron.

**Table 4:** Number of SMN copies and post-treatment effect on the needs for respiratory support.

Author/Year	SMN Copies	Reduced NIV/IMV	Unchanged NIV/IMV
De Holanda Mendonca et al. /2021 [42]	2	4/18	13/18
	3	2/3	1/3
Kim et al. /2020 [39]	2	2/4	2/4
Ogawa et al/2019 [37]	2	0/1	1/1
Tanaka et al/2021 [44]	3	0/1	1/1
Tiberi et al./2020 [41]	1	0/1	0/1
**Total**	**1**	**0/1**	**1/1**
	**2**	**6/23 (26%)**	**16/23 (69.5%)**
	**3**	**2/5 (40%)**	**3/5 (60%)**

Abbreviations: IMV: invasive mechanical ventilation via tracheostomy; NIV: noninvasive ventilation; SMN: survival motor neuron.

**Table 5:** Changes in ventilatory support requirements following treatment with gene therapy.

Author/Year	Reduction in NIV/IMV Support	Discontinued NIV	Unchanged NIV/IMV Support	Criteria for Ventilator Support Changes
D’ Silva et al./2022 [45] *	2/7	0	5/7	Respiratory decompensation
Mateszenz et al./2022 [46] *	0	0	1/1	n/m
Weiß et al./2022 [47] **	9/24	1/24	n/m	Polysomnography
Waldrop et al. /2020 [48] **	1/9	1/9	7/9	n/m
**Total-** **Gene Therapy**	**12/41 (29.2%)**	**2/41 (4.9%)**	**n/a**	**n/m**

Abbreviations: IMV: invasive mechanical ventilation via tracheostomy; NIV: noninvasive ventilation; n/m: not mentioned; n/a: not applicable; * all patients previously had Nusinersen; ** Nusinersen naïve patients were included.

## Data Availability

Not applicable.
